# Porous Polystyrene Monoliths and Microparticles Prepared from Core Cross-linked Star (CCS) Polymers-Stabilized Emulsions

**DOI:** 10.1038/s41598-017-09216-y

**Published:** 2017-08-17

**Authors:** Qijing Chen, Ting Shi, Fei Han, Zihan Li, Chao Lin, Peng Zhao

**Affiliations:** Shanghai East Hospital, The Institute for Biomedical Engineering and Nanoscience, Tongji University School of Medicine, Tongji University, Shanghai, 200092 People’s Republic of China

## Abstract

A hydrophobic CCS polymer of poly(benzyl methacrylate) (PBzMA) was prepared in toluene by reversible addition-fragmentation chain transfer (RAFT)-mediated dispersion polymerization. The CCS polymer, with poly(benzyl methacrylate) as the arm and crosslinked *N*, *N*′-bis(acryloyl)cystamine (BAC) as the core, was confirmed by characterization with gel permeation chromatography (GPC) and nuclear magnetic resonance (NMR) spectroscopy. Three kinds of oils (toluene, anisole and styrene) were chosen to study the emulsification properties of PBzMA CCS polymer. The oils can be emulsified by CCS polymer to form water-in-oil (w/o) emulsions. Moreover, w/o high internal phase emulsions (HIPEs) can be obtained with the increase of toluene and styrene volume fractions from 75% to 80%. Porous polystyrene monolith and microparticles were prepared from the emulsion templates and characterized by the scanning electronic microscopy (SEM). With the internal phase volume fraction increased, open-pore porous monolith was obtained.

## Introduction

An emulsion comprises two phases of the dispersed phase and the continuous phase. It is a thermodynamically unstable system^1^ and demulsification phenomena gradually occur with the time, finally leading to two-phase complete separation. In order to enhance the stability of emulsions, stabilizers or emulsifiers are indispensable. There are two kinds of emulsifiers generally. One is the traditional surfactant consisting of a hydrophilic head (polar) and a hydrophobic tail (non-polar), such as Span 80^2^, SDS^3^ and Tween 80^4^. The other is solid particles and the particle-stabilized emulsions are also called Pickering emulsions^5–7^. The solid particle can irreversibly absorb on the oil-water interface so Pickering emulsions are more stable compared with the surfactant-stabilized emulsions.

With the dispersed phase volume fraction increased above 74%, and if there is no phase inversion, the stable emulsions are called high internal phase emulsions (HIPEs)^8, 9^. Generally, HIPEs need large amounts of the surfactant (5~50%) to keep stable^10, 11^. Compared with surfactant, particles with lower concentration can stabilize Pickering HIPEs because of their high absorption energy. The commonly used particle-emulsifiers include inorganic particles (e.g. SiO_2_^12^, TiO_2_^13^ and Fe_3_O_4_^14^) and organic particles (e.g. polystyrene^15^ and microgels^16, 17^).

Recently, we reported that core cross-linked star (CCS) polymers, which advocate their role as an intermediate between linear polymers and polymeric nanoparticles, can also act as effective interfacial stabilizers for emulsions^18^. Poly(*N*,*N*-dimethylaminoethyl methacrylate) (PDMAEMA)^19, 20^, poly(MEA-*co*-PEGA) (MEA is 2-methoxyethyl acrylate, PEGA is poly(ethylene glycol) acrylate)^21^ and styrenic imidazole-based CCS polymers^22^ are all excellent emulsifiers to stabilize oil-in-water (o/w) emulsions and HIPEs. So far, w/o emulsions and HIPEs has not been formed because most of the CCS polymers are hydrophilic. Even for less hydrophilic styrenic imidazole-based CCS polymer (S-PVBnIm), o/w HIPEs were still formed rather than w/o emulsions.

Emulsions has many applications in cosmetics, pharmaceuticals, food, oil recovery and chemical processing. For HIPEs, one important application is used as template to prepare porous materials^23, 24^. For o/w HIPEs, hydrophilic materials were achieved by polymerizing the hydrophilic monomers in the continuous phase and removing the dispersed oil phase. For the w/o HIPEs, hydrophobic materials can be obtained by polymerization of hydrophobic monomers in the oil and remove of the water.

Herein, in order to broaden the application of CCS polymers in w/o HIPEs, hydrophobic poly(benzyl methacrylate) (PBzMA) CCS polymer was prepared. An group found that both dispersion and emulsion polymerizations are advantageous for CCS preparation^25^. In this article, PBzMA CCS polymer was synthesized for the first time by RAFT-mediated dispersion polymerization in toluene. Three kinds of oils, i.e. toluene, anisole and styrene, were chosen to study the emulsification properties of PBzMA CCS polymer. Emulsion stability was verified by synthesis of porous polystyrene monolith and microparticles.

## Results

**Figure 1:**
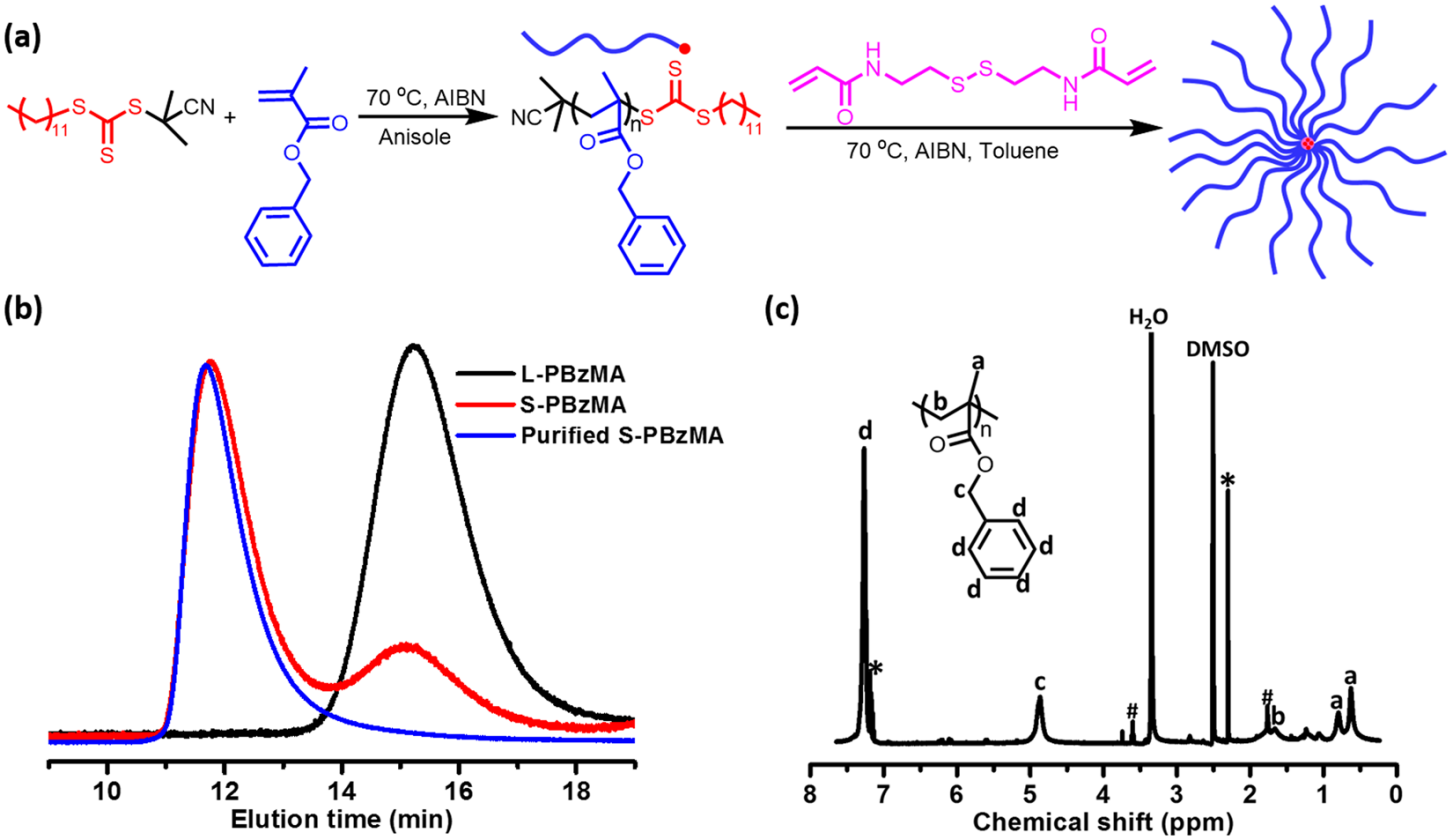
Synthesis of PBzMA CCS polymer. (**a**) The schematic diagram of the synthesis steps. (**b**) GPC trace confirming the formation of the CCS polymer. (**c**) ^1^H NMR spectra of the CCS polymer (recorded in DMSO-*d*_6_).

PBzMA CCS polymer was prepared by the arm-first method *via* RAFT dispersion polymerization in toluene and the synthesis scheme was depicted in Fig. 1a. First, L-PBzMA arm was synthesized *via* RAFT polymerization controlled by *S*-(2-cyanoprop-2-yl)-*S*-dodecyltrithiocarbonate as the RAFT agent and AIBN as initiator at 70 °C in anisole. The polymerization was stopped at 77% monomer conversion to limit the formation of dead polymers. L-PBzMA arm with *M*_n_ of 9.9 kg/mol and *Ð* of 1.15 was achieved and the GPC trace was presented in Fig. 1b. The L-PBzMA arm was then used as the macro-CTA to prepare PBzMA CCS polymer (S-PBzMA) using *N*, *N*′-Bis(acryloyl)cystamine (BAC) as the cross-linker. After polymerization for 48 hours, the crude star polymer was monitored by GPC and the incorporation of arm polymer into the star is above 80%. In order to obtain S-PBzMA with high purity, the as-synthesized crude product was purified by dialysis. From Fig. 1b, the well-defined S-PBzMA with *M*_n_ of 180.1 kg/mol and *Ð* of 1.2 was obtained after purification. The S-PBzMA was characterized by ^1^H-NMR (Fig. 1c). All of the characteristic peaks of the arm of L-PBzMA were found. The core of S-PBzMA was highly cross-linked and thus the protons in the core were not detectable by ^1^H-NMR measurement. There are very little residual solvents (* represents hexane and # represents tetrahydrofuran) in the stiff solid which have no effects on the study of the emulsification properties of the CCS polymer.

**Figure 2:**
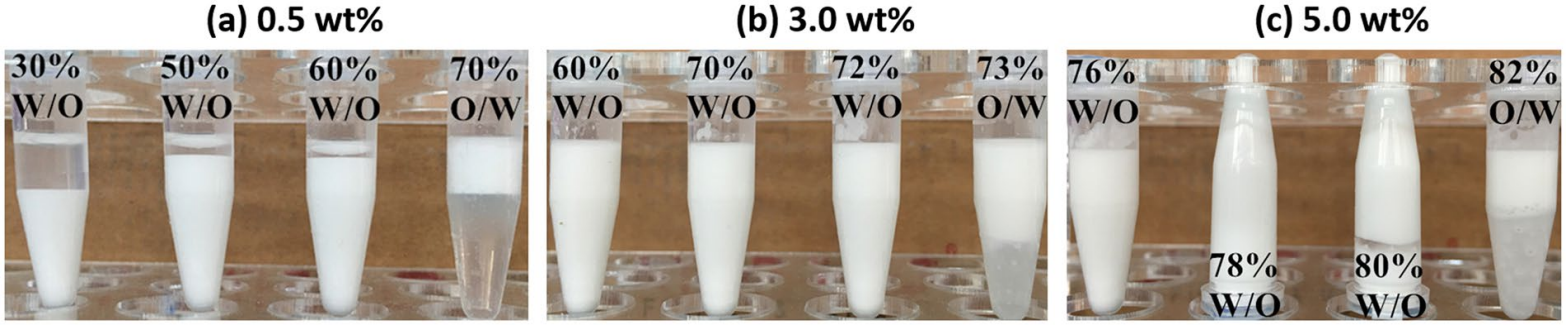
Photographs of emulsions with toluene as the oil stabilized by S-PBzMA with different concentrations of (**a**) 0.5 wt%, (**b**) 3.0 wt%, and (**c**) 5.0 wt%, respectively. The percentage number on the vial represents the water volume fraction (*V*_w_).

**Figure 3:**
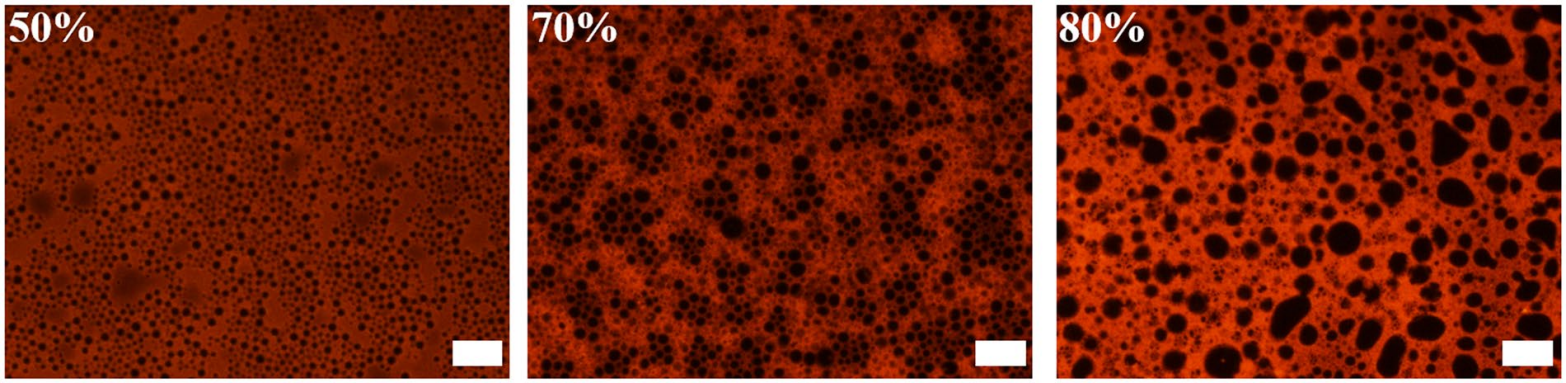
Fluorescence microscopy of emulsions stabilized by 5.0 wt% S-PBzMA with styrene as the oil. The percentage numbers are *V*_w_ of the emulsions and the scale bars are all 50 *μ*m.

S-PBzMA is highly hydrophobic and can dissolve in most of the organic solvents. In order to research the emulsification property of the star polymer, three common organic solvents (i.e. toluene, anisole and styrene) were chosen as the oil phase. All of the emulsions were prepared in the vials of 1.5 mL and the total emulsion volume was 1 mL. For toluene, when the emulsifier concentration is 0.5 wt%, the critical *V*_w_ above which emulsion type inverted from w/o to o/w emulsion is between 60% and 70% (Fig. 2a). Herein, dilution method was used to identify the emulsion type. If the emulsion was miscible with the water, the emulsion type was o/w. If not, it was w/o. With the increase of emulsifier concentration, the critical *V*_w_ increased. For 3.0 wt% and 5.0 wt% PBzMA CCS polymers, the critical water volume fractions are 72% and 80%, respectively (Fig. 2b and c). So the CCS polymer is inclined to stabilize toluene to form w/o emulsions at high concentrations. For *V*_w_ of 78% and 80% in Fig. 3c, emulsions lost their fluidities and formed w/o HIPEs. In addition of toluene, S-PBzMA can also stabilize styrene to form w/o HIPEs. However, for anisole, when the *V*_w_ was above 50%, the emulsions inverted from w/o to o/w at any concentrations of the emulsifier. The ether bond of the anisole was the main reason why the emulsion possessed the low critical *V*_w_. In addition, conductivity measurement of emulsions was done to identify emulsion type as a complementary means. For the w/o emulsions, the conductivities were all zero. For the o/w emulsions, the conductivities distributed in the range of 28~63 *μ*s/cm (Table S1).

Fluorescence microscopies further verified that the emulsion type was w/o. The oil phase was dyed by Nile red. When the *V*_w_ increased from 50% to 80%, the emulsions were all w/o types. With the *V*_w_ increased, the sizes of water droplets increased. The water droplet diameters were in the range of 5~10 *μ*m for the emulsion with *V*_w_ = 50%, and 10~20 *μ*m for the emulsion with *V*_w_ = 70%. Indifferent from the emulsions with *V*_w_ of 50% and 70%, the water droplets for the emulsion with *V*_w_ of 80% are more nonuniform of 10~50 *μ*m. The bigger droplets around 50 *μ*m is due to the insufficient shocking by the device. The emulsions were prepared by violently shaking the vial back and forth. Droplet sizes became smaller with the time^26^. For the emulsions with low *V*_w_, the emulsion droplets can obtain complete smash by high speed flowing with the vibrating device. Uniform and small droplets were achieved at last. When the *V*_w_ is 80% or more, emulsion formed at the beginning of the shaking was very viscous and would be more viscous with the time. Finally, the shaking cannot further smash the droplets because the viscous emulsion lost its fluidity. Therefore, some big droplets were packed in the emulsion and some of them were not spherical. This phenomenon was similar with the before research in which emulsions were prepared by high speed shear^27^. The viscoelasticity of the emulsions was further quantitatively investigated by rheology. Figure S2 shows storage modulus (*G*′) and loss modulus (*G*′′) of these emulsions as a function of the frequency at a fixed strain of 1.0%. It was found that the *G*′ increased with increasing *V*_w_ from 50% to 80%, implying augmented viscoelasticity of the emulsions as a result of enhanced accumulation density of emulsion droplets.

**Figure 4:**
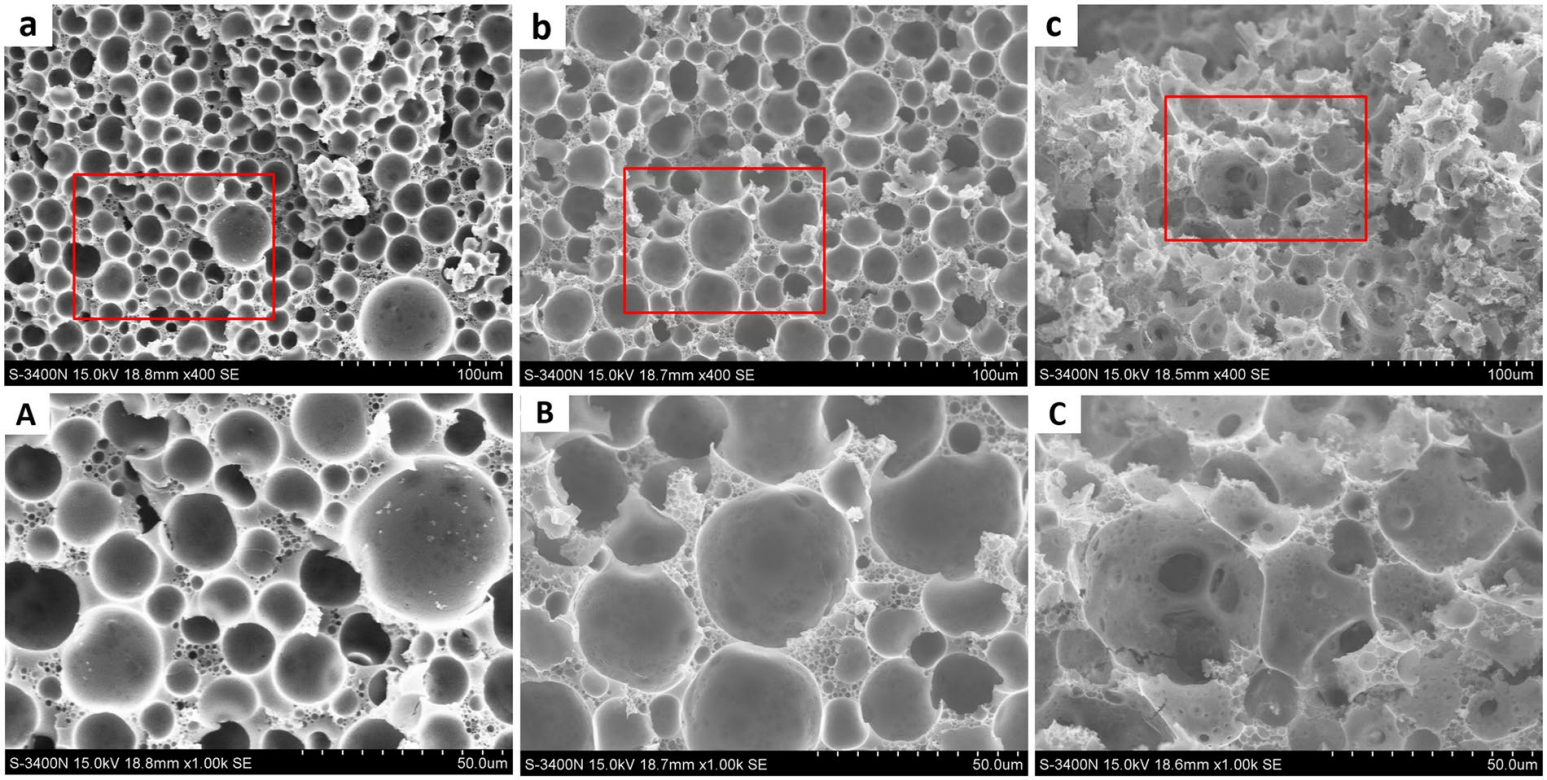
SEM photos of polystyrene monoliths prepared from emulsions stabilized by 5 wt% S-PBzMA polymer with (**a**) *V*_w_ = 50%, (**b**) *V*_w_ = 70% and (**c**) *V*_w_ = 80%, respectively. The A, B and C are the enlarged images of red boxes in (**a**,**b** and **c**) respectively.

To verify the stability of the emulsions stabilized by PBzMA CCS polymer, porous materials were prepared based on the emulsion templates. The pre-prepared styrene with the inhibitor removed was as the continuous phase. After the emulsifier of S-PBzMA polymer and initiator of AIBN were completely dissolved into the styrene, the solution was bubbled by nitrogen. Emulsions with *V*_w_ of 50%, 70% and 80% were prepared and then placed in high temperature for polymerization. After removed the dispersed phase of water by freeze-drying, polystyrene porous monoliths were obtained. The microstructures of the monoliths were observed by SEM (Fig. 4). The monoliths were all very porous and the void sizes increased with the *V*_w_ rising. For the emulsion-templates with *V*_w_ of 50%, 70% and 80%, the void sizes were around 15 *μ*m, 25 *μ*m and 40 *μ*m respectively except for few large voids. The void sizes for *V*_w_ of 50% and 70% were a little bigger than the droplet sizes as shown in fluorescence microscopies (Fig. 3). The emulsions with *V*_w_ of 50% and 70% were not HIPEs and possessed fluidity at the high-temperature polymerization, which led to emulsion instability. Coalescence among emulsion droplets occurred in the high temperature of 70 °C and generated large holes in the poly-emulsions. For the HIPEs with *V*_w_ of 80%, viscosity of the emulsion strengthened the polymerization stability and no bigger holes than the droplet size can be seen. From the enlarged views, three size populations can be seen. One is the voids templated by the droplets in emulsion and the second is the interconnecting pores in the wall resulting from breakdown of the thin wall during solvent extraction process. The number of pores in the wall increased with the *V*_w_ rising of the emulsions. The third pores centralized between the wall mainly for poly-emulsions of *V*_w_ = 50% and 70%, and the sizes were below 5 *μ*m. These pores were produced by unreacted styrene volatilizing in the dried process.

**Figure 5:**
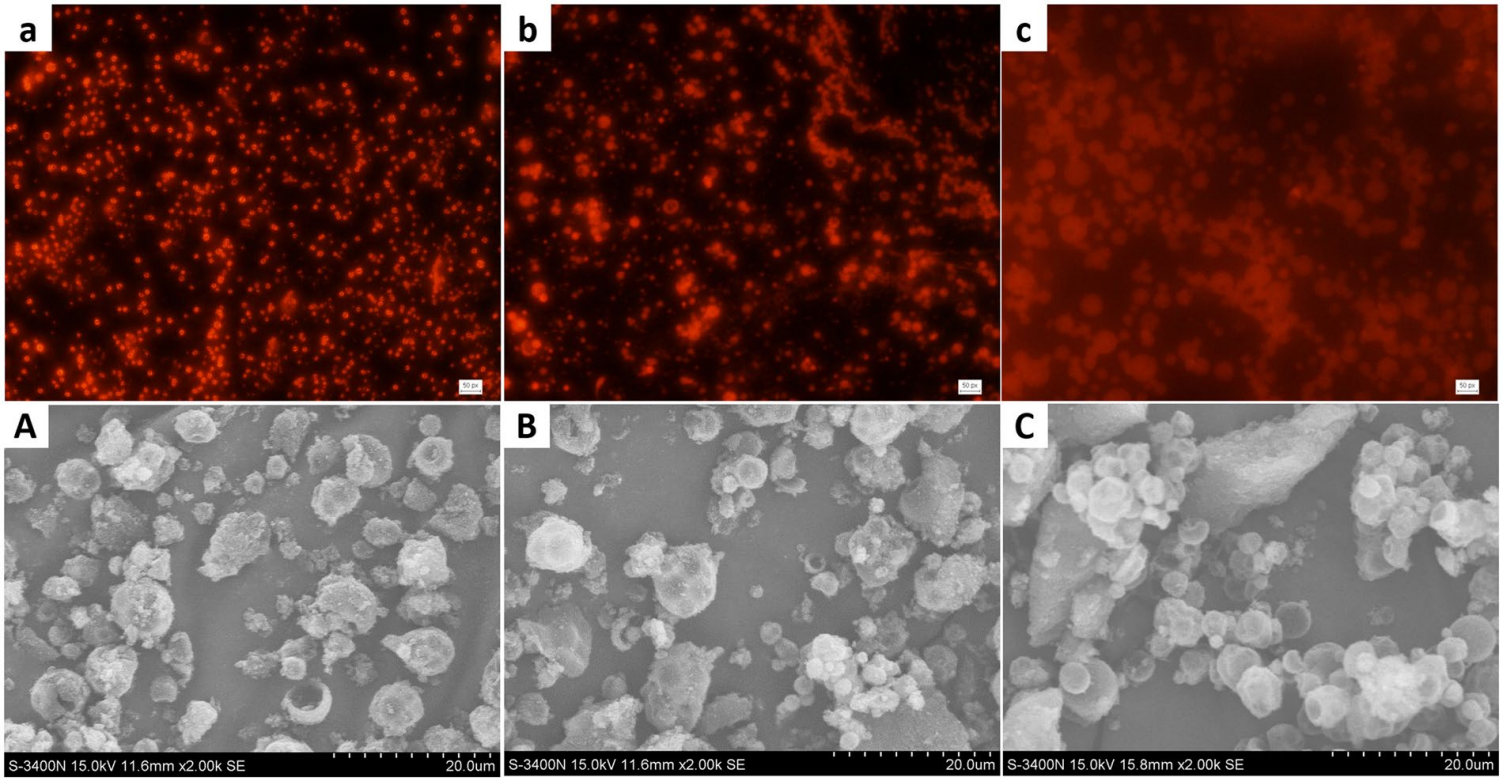
Fluorescence microscopies of the w/o/w multiple emulsions with different oil volume fractions of (**a**) 80%, (**b**) 50% and (**c**) 30% and the oils are styrene. All of the scale bars are 50 *μ*m. A, B and C are the SEM photos of the polystyrene particles prepared from the corresponding multiple emulsions.

Water-in-styrene emulsions were added in the water to prepare w/o/w multiple emulsions. Emulsions were dispersed well in the water from the fluorescence microscopies (Fig. 5) and the sizes of the emulsion droplets in water increased with the oil volume fraction (*V*_o_) decreased. The viscosity of the w/o emulsions increased with the *V*_o_ decreased, so it is comparatively hardest to smash the w/o emulsion with *V*_o_ of 30% and the emulsion droplet size was the biggest. Polymerizing the multiple emulsions at high temperature, styrene-based particles were obtained. The microstructures of the particles were observed by SEM. The particle sizes distributed in the range of 2~6 *μ*m. The polystyrene material was hard and the polystyrene-based porous particles were all unbroken (Figure S2). In order to judge if the particles were porous, the grinded polystyrene particles for SEM were shown in Fig. 5. The sizes of the particles were slightly smaller than the corresponding multiple emulsions because of the shrink of the polystyrene in the polymerization. There were several particles broken by grinding and the microparticles were hollow judged from the broken particles. In addition, some of the microparticles were depressed deformation which indicated that these particles were hollow. However, some particles aggregated together because of the instability of the multiple emulsions.

## Discussion

In previous work, hydrophilic CCS polymers were prepared in water^25^ or water/ethanol solutions^28^ by heterogeneous emulsion or dispersion polymerization. However, the arm of L-PBzMA was neither water nor ethanol soluble. In our study, we find that L-PBzMA polymer can be dissolved well in toluene. So, if a crosslinker with a low solubility in toluene is chosen, PBzMA CCS polymer should be prepared by dispersion polymerization in toluene. Boyer *et al*. found that *N*, *N*′-bis(acryloyl)cystamine (BAC) had lower solubility in toluene compared with *N*,*N*-methylene bisacrylamide, 2-dihydroxyethylene-bis-acrylamide and 1,6-hexanediol diacrylate^29^. Thus, BAC is chosen to extend chain at a [L-PBzMA]/[crosslinker]/[AIBN] ratio of 1/8/0.2. After polymerization for 48 hours, the incorporation of arm polymer into the star at above 80% is achieved. Therefore, well-defined PBzMA CCS polymer can be prepared by RAFT-mediated dispersion polymerization in toluene.

Emulsion type is associated with the amphiphilicity of the emulsifier^30^. Hydrophilic emulsifiers prefer to stabilize o/w emulsion and hydrophobic emulsifiers tend to form w/o emulsion. Generally, an emulsion tends to be phase inversion with the increase of the dispersed phase volume fraction, and the critical volume fraction is normally 50%. Most of stable HIPEs are produced in relatively hydrophobic liquids. Moreover, to form HIPEs^8^, the emulsifier should be completely insoluble in dispersed phase of the emulsion. As such, rational choice of emulsifier and the organic phase can prevent phase inversion to form HIPEs. In order to broaden the application of CCS polymer in w/o HIPEs, poly(benzyl methacrylate) is chosen as the hydrophobic arms and crosslinked BAC as the core. Both the arm and core are so hydrophobic that the CCS polymer prefers to form w/o emulsions and HIPEs. The S-PBzMA polymer is well soluble in toluene, styrene and anisole but completely insoluble in water. The o/w HIPEs can be produced for toluene and styrene but anisole, because toluene and styrene were more hydrophobic compared with the anisole. The ether bond of the anisole weakens its hydrophobicity and the emulsions with the anisole invert from w/o to o/w.

**Figure 6:**
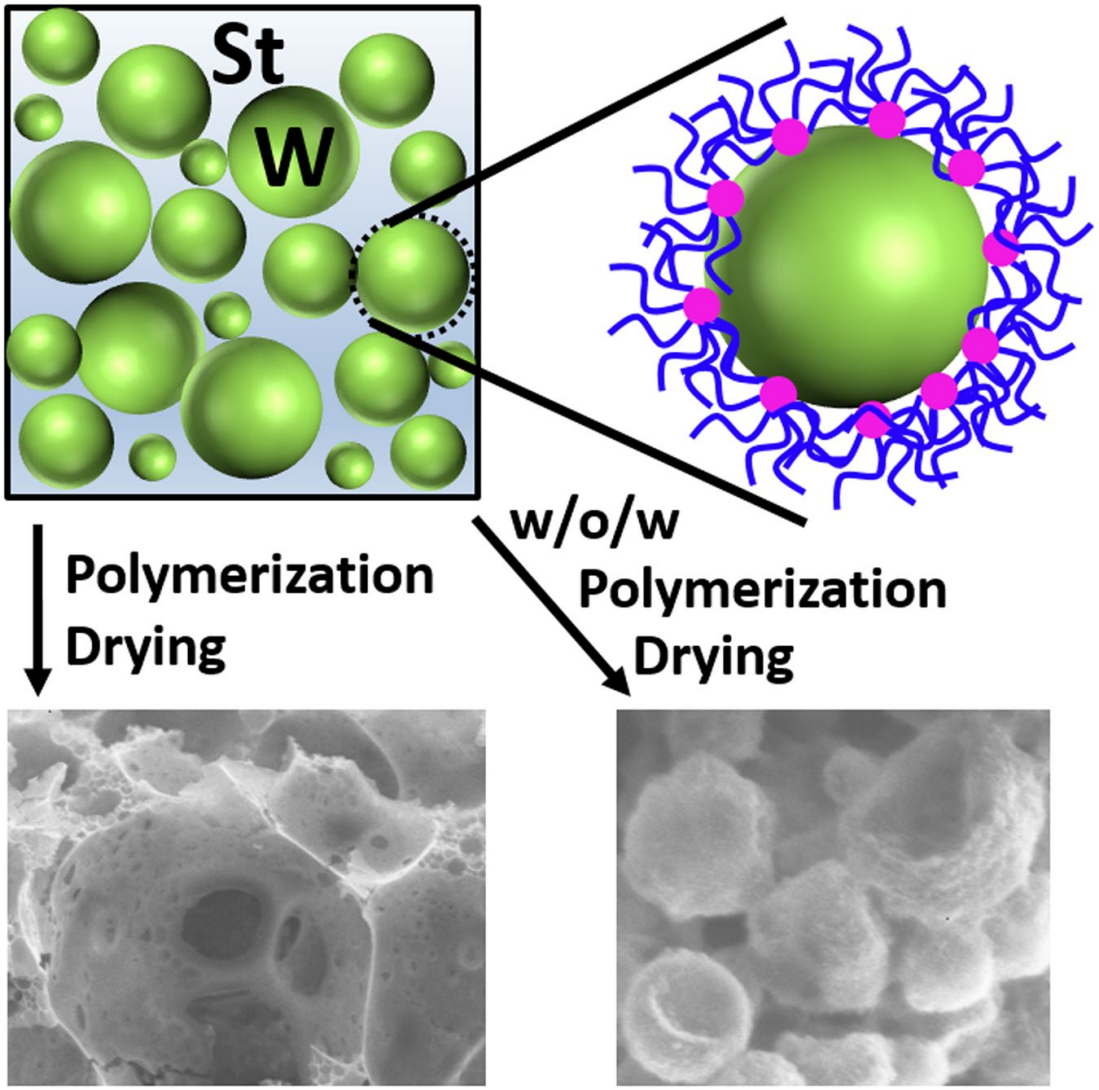
Schematic representation of styrene porous materials prepared from PBzMA CCS polymer-stabilized emulsions.

Matyjaszewski *et al*.^31^ studied the emulsification properties of poly(ethylene oxide) (PEO)-based star polymers and found that enhancing hydrophobicity of the star polymer by introducing hydrophobic arms into the star can change the emulsion type from o/w to w/o. The emulsification mechanism was also proposed. For the o/w emulsions, the hydrophobic core adsorbed on the oil/water interface and the hydrophilic arms of PEO dispersed in the water. For the w/o emulsion, the hydrophobic core adsorbed on the oil/water interface and the hydrophilic arms of PEO stayed in the water and the hydrophobic arms of poly(butyl acrylate) (PBA) dispersed in the oil phase. In our study, the hydrophobic arms of L-PbzMA have no solubility in water and would stay in the oil phase. The cross-linker BAC has low solubility in styrene, and after the polymerization crosslinking, its solubility may further decrease. The BAC also has low solubility in water. So the core tends to stay in the interface like a particle to low interfacial tension. For styrene-in-water emulsion, the emulsification scheme is shown in the Fig. 6. The long arms in the continuous phase intertwined with each other strengthen the stability of the emulsions. The emulsification mechanism is consistent with the previous studies of o/w HIPEs stabilized by CCS polymers^18–22^.

Porous materials of polyHIPEs, prepared with HIPEs templates, have two kinds of structures of open pore and closed pore^32^. Closed-pore materials have only one kind of voids produced by the removal of the dispersed phase. For open-pore materials, except the voids templated by emulsion droplets, there is other hole in the wall connecting the adjacent voids. Therefore, an open-pore structure possesses higher permeability compared with a closed-pore structure. Generally, surfactant-stabilized HIPEs produce open-pore materials. However, a high concentration of surfactant (5~50 vol%) often limits its broad applications^33^. Pickering HIPEs decreased the use of particles, however, poly-Pickering HIPEs commonly had a closed-pore structure^34^. Before, we prepared a porous hydrogel based on the o/w HIPEs stabilized by PDMAEMA CCS polymer^19^. The hydrogel was closed-pore structure because the material was so soft that holes in the wall collapsed in the process of drying and re-swelling. Herein, polystyrene material was relatively hard. With *V*_w_ increased from 50% to 80%, the wall thickness gradually decreased and holes in the wall appeared (Fig. 4c). Porous styrene monolith with an open-pore structure was produced based on CCS polymer stabilized HIPEs. The CCS polymer has a number of arms connected to a central core, which is smaller in size than the dimension of the arms. In an emulsion, the core of the CCS polymer absorbed on the oil/water interface, and the arms dispersed and intertwined with each other in the continuous phase. The core of the CCS polymer cannot tightly arrange on the interface with high density like particles due to the arms obstruction. The linear arms act as the surfactant when polymerization of the continuous phase, resulting in the formation of small pore throats in the wall.

Polystyrene porous particles were prepared from the w/o/w multiple emulsions. The w/o/w multiple emulsion prepared by directly dispersing the w/o emulsion into the water was unstable enough in the polymerization at high temperature because there was no stabilizer in the water. So there are some aggregations of the particles.

In summary, poly(benzyl methacrylate) CCS polymer can be obtained by RAFT-mediated dispersion polymerization in toluene. PBzMA CCS polymer may serve as a useful emulsifier to stabilize organic liquids such as toluene and styrene, forming w/o HIPEs. Based on the emulsion templates, porous polystyrene monoliths with open pores and porous polystyrene microparticles can be fabricated successfully. This is the first report on w/o HIPEs stabilized by CCS polymer.

## Methods

### Materials

Benzyl methacrylate (BzMA, 96%), *S*-(2-cyanoprop-2-yl)-*S*-dodecyltrithiocarbonate (CTA, 97%), styrene (99%) and Nile red (98%) were purchased from Sigma-Aldrich. Tetrahydrofuran (THF, 99+%), toluene (CP), anisole (AR), hexane (AR) were purchased from Sinopharm Chemical Reagent. 2,2′-Azobis(2-methylpropionitrile) (AIBN, 99%) was purchased from Aladdin Reagent. *N*, *N*′-Bis(acryloyl)cystamine (BAC, 98%) was purchased from Alfa-Aesar. All liquid monomers were passed through a column of Al_2_O_3_ to remove the inhibitor prior to use.

### Characterization

Polymer structures were characterized by NMR spectra collected on a Bruker AV 500 MHz spectrometer in DMSO-*d*_6_ and the chemical shifts were reported using the solvent residue as the reference. Molecular weight and polydispersity index of polymer were measured by gel permeation chromatography (Viscoteck Instrument, Malvern, UK) equipped with one MBMMW column and Triple Detection System (S100, Malvern, UK), using DMF/LiBr (0.02 M) as an eluent at flow rate of 1 mL/min. Fluorescence microscopy was performed on an inverted phase contrast fluorescence microscope (Olympus IX 70) using the blue light to excite the samples stained with Nile red in the oil phase. The samples were directly placed on the slides. Photographs of emulsions were taken by digital camera (iphone 6 S). SEM was performed on a Hitachi S-3400 microscope and the samples were coated with platinum. Conductivity of emulsions was measured using Mettler Toledo device equipped with an ISM electrode. Rheological measurements were performed on a HAAKE MARS III rotary rheometer at 25 °C using the plate geometry.

### Synthesis of PBzMA macro-CTA (L-PBzMA)

PBzMA macro-CAT was prepared by RAFT polymerization^35^. CTA (0.520 g, 1.46 mmol) and BzMA monomer (16.087 g, 87.60 mmol) were dissolved in 35 mL of anisole. The solution was degassed with nitrogen at 0 °C for 40 min before immersion into a preheated oil bath at 70 °C. After the temperature was stabilized, a degassed solution of AIBN (0.048 g, 0.15 mmol) in anisole was injected *via* a microsyringe. The polymerization was conducted for 6 h and was stopped at 77% monomer conversion as determined by ^1^H NMR. The solution was precipitated into hexane for purification. The precipitation was re-dissolved by THF and precipitated into hexane again and the purification process was done for three times until the unreacted monomer was removed completely. After drying under vacuum,12.12 g of a yellow solid was obtained in 73% yield. *M*_n,th_ = 8.5 kg mol^−1^, *M*_n_ = 9.9 kg mol^−1^ (RI-GPC), *Đ* = 1.15 (RI-GPC).

### Synthesis of PBzMA CCS polymer (S-PBzMA)

PBzMA CCS polymer was synthesized by RAFT-mediated dispersion polymerization in toluene. PBzMA macro-CTA (8.049 g, 0.95 mmol), BAC (3.036 g, 11.43 mmol) and AIBN (0.032 g, 0.19 mmol) were dissolved in toluene of 160 mL. After degassed with nitrogen in an ice/water bath for 40 min, the mixture was immersed into a preheated oil bath at 70 °C. The polymerization was allowed to continue for 48 h under protection of nitrogen. The yield of the star polymers was above 80% calculated from GPC chromatograms (Star yield = area of star polymer/(area of star polymer + area of low molecular weight polymer). RI-GPC: *M*_n_ = 180.1 kg mol^−1^, *Đ* = 1.2.

### Preparation of emulsions

The total volume of all the prepared emulsions was 1 mL and the water phase volume fraction (*V*_w_) was varied from 30% to 85%. The oils include toluene, anisole and styrene. The CCS polymer was dissolved in the oil with different concentrations. An organic solution of the CCS polymer was mixed with water, and the mixture was homogenized using a Mini-Beadbeater^TM^ (OA60AP-22-1 WB) for 3 minutes. The emulsion type was identified by fluorescence microscopy with the emulsions stained by Nile red in the oil phase.

### Preparation of styrene porous monoliths

PBzMA CCS polymer was dissolved into styrene as the oil phase and CCS concentration is 5 wt%. The initiator of AIBN was dissolved into the oil phase with the concentration of 0.5 wt%. Then the oil phase was degassed with nitrogen for 20 min. The emulsions with *V*_w_ of 50%, 70% and 80% were prepared in the vials and then placed into an oven at 70 °C for 24 h. After the polymerization, the vials were freeze dried to get the porous monoliths.

### Preparation of styrene porous microparticles

First, water-in-styrene emulsions (w/o) was prepared as above. Then multiple emulsions of w/o/w were prepared by homogenizing the w/o emulsion and water for 5 s. The emulsion volume fraction (*V*_m_) of the multiple emulsion is 20%. The w/o/w emulsions were placed in the oven at 70 °C for 24 h. After that, the emulsions were freeze dried to get the porous microparticles.

## Electronic supplementary material

Supplementary Information
