# Supplementary material for: Porous Polystyrene Monoliths and Microparticles Prepared from Core Cross-linked Star (CCS) Polymers-Stabilized Emulsions
Source: Sci Rep. 2017 Aug 17;7:8493. doi: 10.1038/s41598-017-09216-y (PMC5561027; doi:10.1038/s41598-017-09216-y)
Supplement: Supplementary file 1 — Supplementary Information [file 41598_2017_9216_MOESM1_ESM.docx]

**Supporting Information**

**Porous Polystyrene Monoliths and Microparticles Prepared from Core Cross-linked Star (CCS) Polymers-Stabilized Emulsions**

**Qijing Chen, Ting Shi, Fei Han, Zihan Li, Chao Lin and Peng Zhao^*^**

Shanghai East Hospital, The Institute for Biomedical Engineering and Nanoscience, Tongji University School of Medicine, Tongji University, Shanghai 200092, People’s Republic of China

E-mail: zp@tongji.edu.cn

**Table S1.** Summary of conductivity measurement of emulsions.

| Oil category | S-PBzMA concentration (wt%) | *V*_w_*^a^* | Conductivity (*μ*s/cm) | Emulsion type |
| --- | --- | --- | --- | --- |
| styrene | 0.5 | 70% | 62.7 | o/w |
| styrene | 3.0 | 73% | 62.1 | o/w |
| styrene | 5.0 | 80% | 0 | w/o |
| styrene | 5.0 | 82% | 62.3 | o/w |
| toluene | 5.0 | 80% | 0 | w/o |
| toluene | 5.0 | 82% | 28.6 | o/w |
| anisole | 5.0 | 50% | 0 | w/o |
| anisole | 5.0 | 60% | 48.5 | o/w |
| *^a^*Water volume fraction of the emulsion. | | | | |

**Figure S1.** Changes in storage modulus, *G*′, and loss modulus, *G*′′, for emulsions (*V*_w_ = 50%, 70% and 80%) stabilized by S-PBzMA of 5.0 wt% during frequency sweeps at a fixed strain of 1.0%. Measurement temperature was 25 °C.

**
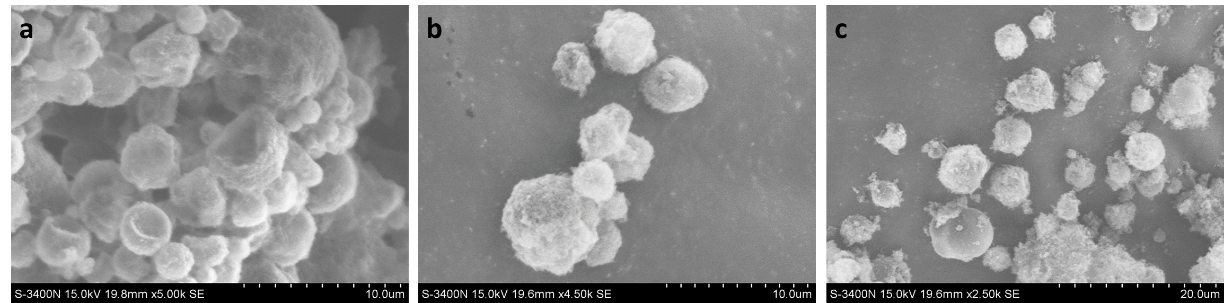
**

**Figure S2.** SEM photos of the polystyrene particles (without grinding) prepared from the w/o/w multiple emulsions with different oil volume fractions of (a) 80%, (b) 50% and (c) 30% and the oils are styrene.
